# Prosthetic Rehabilitation of Narrow Mandibular Ridge by Two-Stage Split Technique: A Case Report

**DOI:** 10.7759/cureus.52764

**Published:** 2024-01-22

**Authors:** Yashika Bali, Benny Budhwar, Ravpreet Singh, Peter Devadoss

**Affiliations:** 1 Department of Prosthodontics and Crown and Bridge, Subharti Dental College and Hospital, Swami Vivekanand Subharti University, Meerut, IND; 2 Department of Orthodontics and Dentofacial Orthopedics, Bhojia Dental College and Hospital, Baddi, IND; 3 Department of Prosthodontics, Baba Jaswant Singh Dental College and Hospital, Ludhiana, IND; 4 Department of Dentistry, Army Dental Center Research and Referral (R&R), Delhi, IND

**Keywords:** two-stage ridge split, bone ridge, implant, bucco-lingual dimensions, missing tooth

## Abstract

Subsequent to dental extraction, residual ridge resorption manifests as an inherent biological process unfolding over an approximate duration of one year. This intrinsic phenomenon entails a substantial diminution, occasionally reaching 50%, in the initial bucco-lingual dimensions of the mandibular bone. To address this issue, a dental procedure known as the two-stage ridge split intervention is employed. This process involves two distinct stages: ridge splitting and extension. In the first stage, the dentist splits the alveolar crest to create a widening gap. This allows for the subsequent placement of dental implants. The splitting process is carefully executed to ensure that there's enough space for the implants to be securely embedded, and in the second stage, the widened gap generated through the split and extension of the alveolar crest is replenished with a suitable material. Two common options are hydroxyapatite, a synthetic bone-like substance that promotes bone regeneration, or autogenous bone grafts, which are harvested from the patient's bone, often from another site within the mouth. Following this two-stage procedure, the next step is to place dental implants. However, there's typically a waiting period of eight to 12 weeks. This interval allows for proper healing and integration of the grafted or filled material with the existing bone before the implants are installed. In this case report, a specific patient's experience with the two-stage ridge split procedure in the mandibular region is mentioned. Such case studies are valuable in assessing the success and viability of this dental intervention in narrow mandibular-width cases.

## Introduction

After tooth extraction, it is often seen that the alveolar ridge has a reduction in size, resulting in a decrease of around 50% in its bucco-lingual dimensions during a one-year period [1]. The aforementioned reduction can produce difficulties due to the limited quantity of bone needed to ensure the accurate placement and alignment of dental implants [2]. As a way to mitigate the limited width of the edentulous bone crest, several approaches can be utilized, such as bone graft blocks, guided bone regeneration, horizontal distraction osteogenesis, and the integration of titanium devices [3]. An appropriate quantity of bone is an essential requirement for the prolonged viability of the placed dental implants. In circumstances where the ridges are exceptionally narrow, it becomes imperative to perform augmentation surgery as an essential intervention. The ridge split approach is employed as a technique for addressing the issue of narrow ridges that possess sufficient height by increasing their breadth [4]. The approach initially known by the term "extension plasty" was developed by Osborn in 1985 [5]. This treatment consists of two stages, with the first stage involving the division and expansion of the alveolar ridge. Following this, the area that has been formed is filled through the utilization of substances such as hydroxyapatite or autogenous bone grafts from the patients themselves. The customary time frame for scheduling the surgical insertion into the dental implant is approximately 8-12 weeks following the first surgical intervention. In conditions with enough height relative to the crest bone but limited width for implant implantation, bone splitting presents itself as a suitable treatment that enables transverse augmentation of the crest. The aforementioned treatment has the potential to obtain successful outcomes through different approaches that do not include the utilization of membranes or bone fillers [6]. In the current case report, we put forward a clinical case report detailing the successful completion of a two-stage ridge split procedure on the mandibular ridge.

## Case presentation

**Figure 1:**
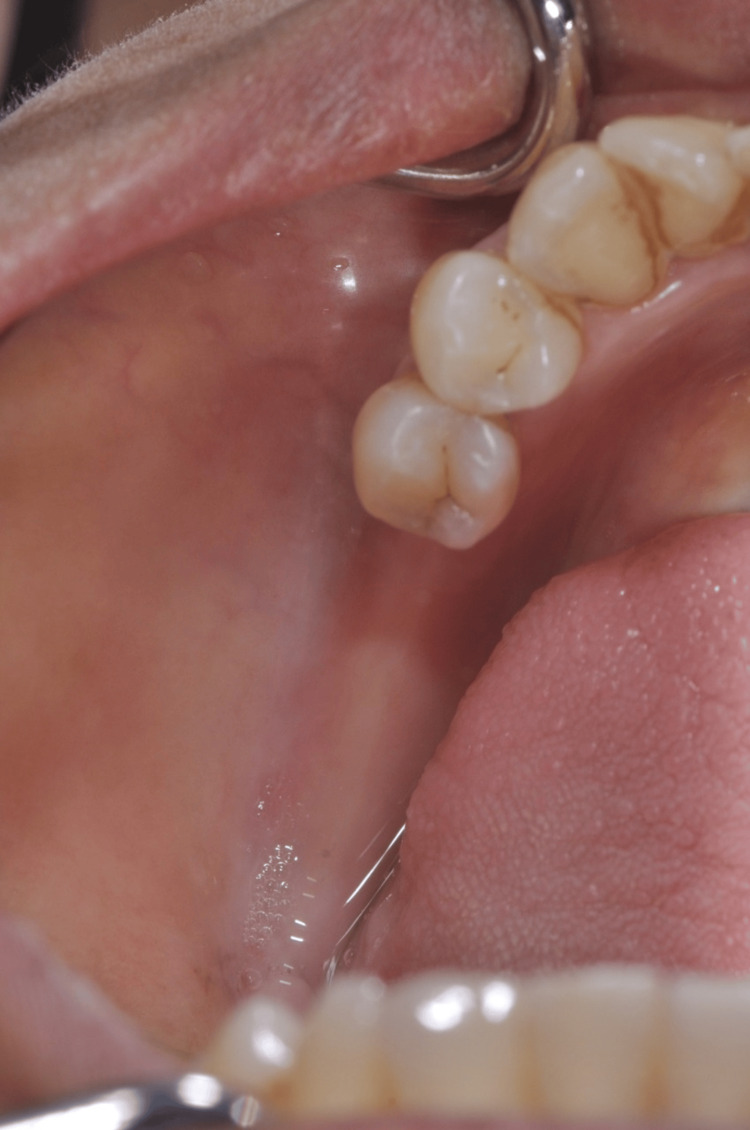
Edentulous ridge in the posterior region with missing teeth (35, 36, and 37)

A male patient, aged 42, reported to the clinic with a two-year history of missing teeth in the lower back tooth region. According to the history given by the patient, it was found that the individual experienced the loss of his lower posterior teeth (35, 36, and 37) around one year ago as a consequence of an accident. The patient wants prosthetic teeth in the same location. Comprehensive oral examination revealed narrow edentulous ridges in the mandibular posterior region with the absence of teeth (35, 36, and 37) (Figure 1), which was further confirmed by extensive radiographic evaluation through CT.

**Figure 2:**
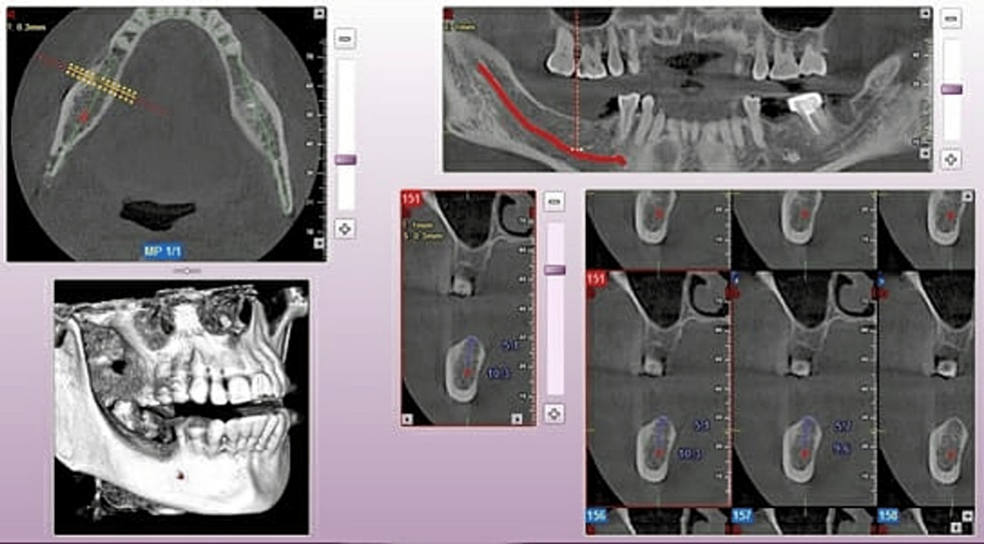
Pre-operative computer tomography (CT) image

The pre-operative CT images revealed a narrow mandibular ridge in the posterior region (Figure 2). Treatment options were discussed with the patient: either a fixed partial denture or implants. Since the patient was not ready to compromise healthy tooth structure and was convinced of implants, we decided to perform a two-stage ridge split procedure through the mandibular ridge.

**Figure 3:**
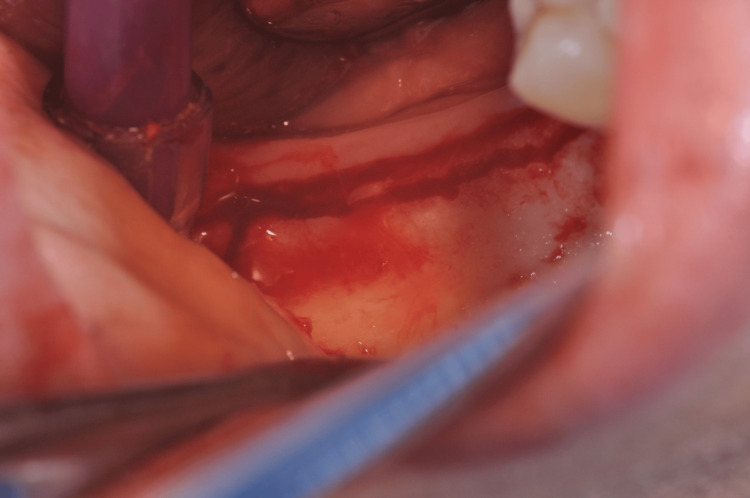
Surgical incision around the edentulous area and neighboring teeth

The patient was informed regarding the procedure, and informed consent was obtained before the surgical procedure. The patient was prepared according to the standard sterile protocol. A loading dose of amoxicillin (1 mg IV) was given one hour before the procedure. The inferior alveolar nerve was anesthetized with a 3% concentration of mepivacaine in combination with a 4% articaine solution, and epinephrine 1/100,000 was administered. The surgical approach entailed making an incision with a 15-number blade, following a lingual trajectory next to the alveolar ridge in edentulous areas. A sharp palatal to mid-crestal incision was given to raise a full-thickness flap that encircled the neighboring teeth, reaching approximately 3-4 mm in distance (Figure 3).

**Figure 4:**
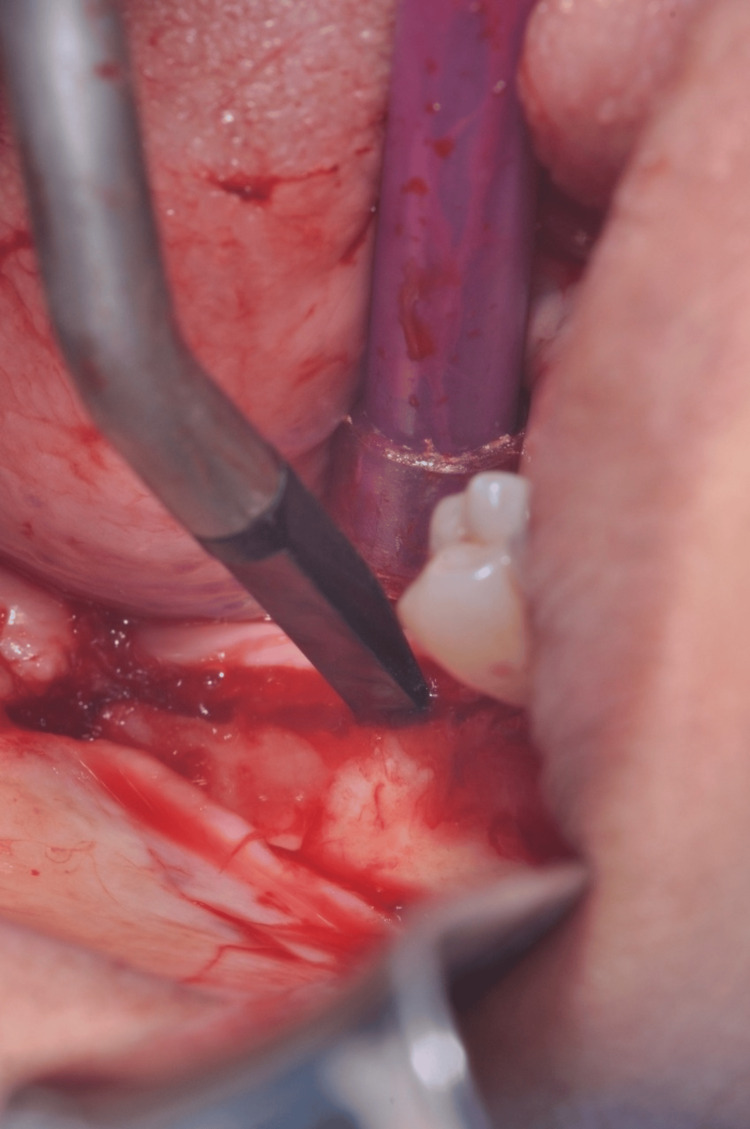
Flap elevation and exposure of buccal bone surface by using a chisel

The objective of the surgical step was to safeguard the papillae while preserving the quality of periodontal connection to the neighboring regions. A thin layer, originally of restricted thickness, was raised to the border between the mucosa and the gingiva. Following that, the surgical cut proceeded to penetrate farther, revealing the outside surface that covered the buccal bone, resulting in the complete depth of the flap (Figure 4).

**Figure 5:**
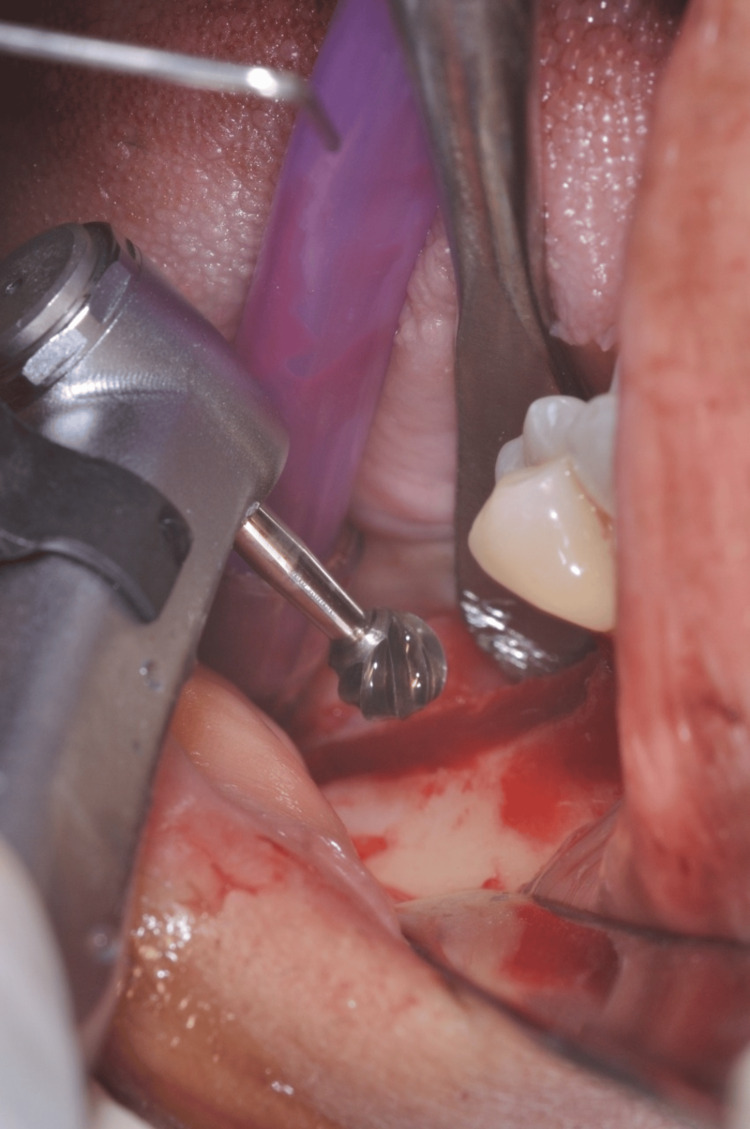
Corticotomy and extension of keratinized mucosal coverage

A horizontal corticotomy was performed using micro-saws attached to a sonic air surgical instrument. Starting from the extremities of this horizontal incision, two vertical corticotomies were continued coronally, reaching the mucogingival line. Subsequently, flaps were apically repositioned with interrupted suturing with 3.0 silk (Ethicon Mersilk, Ethicon Inc., Raritan, New Jersey) and anchored at the periosteum layer, therefore increasing the extent of keratinized mucosal covering (Figure 5).

**Figure 6:**
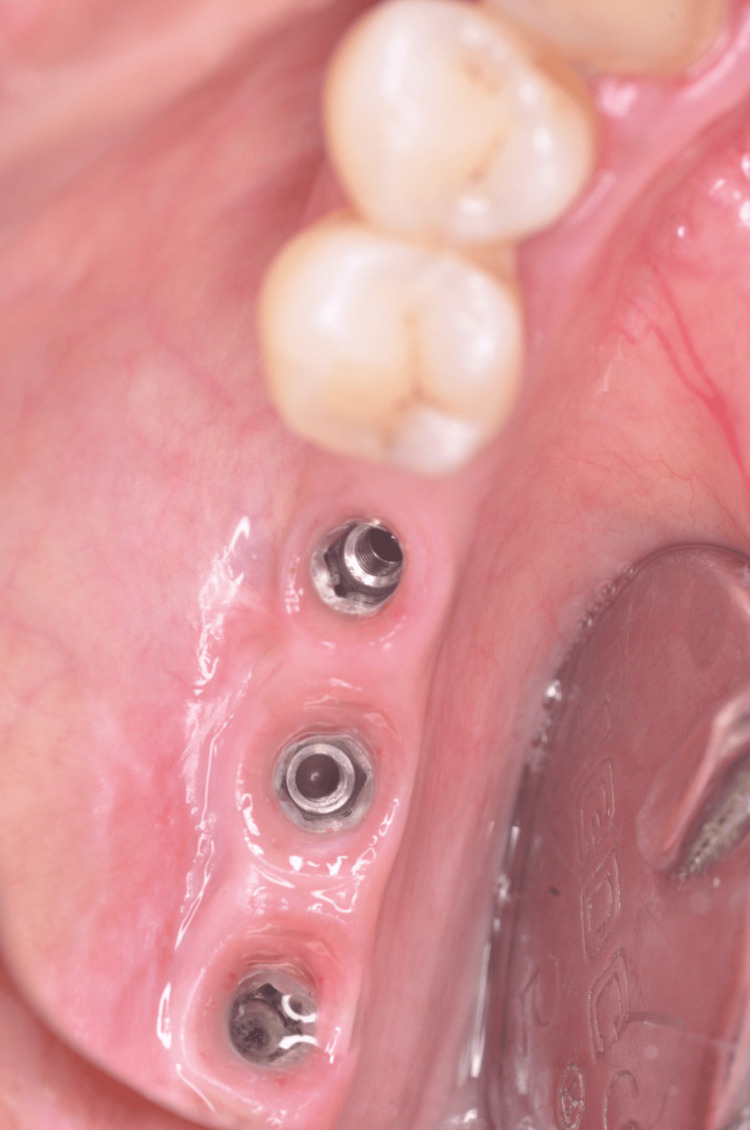
Implant placement in buccal and lingual bone

The subsequent procedures involved the implementation of sagittal and transverse osteotomies, with the specific objective of mobilizing the buccal bone section of the plate. Subsequently, three implants (3.75 mm in diameter x 11.5 mm in length) were carefully inserted into the interstices that demarcate the buccal and lingual bone boundaries (Figure 6).

**Figure 7:**
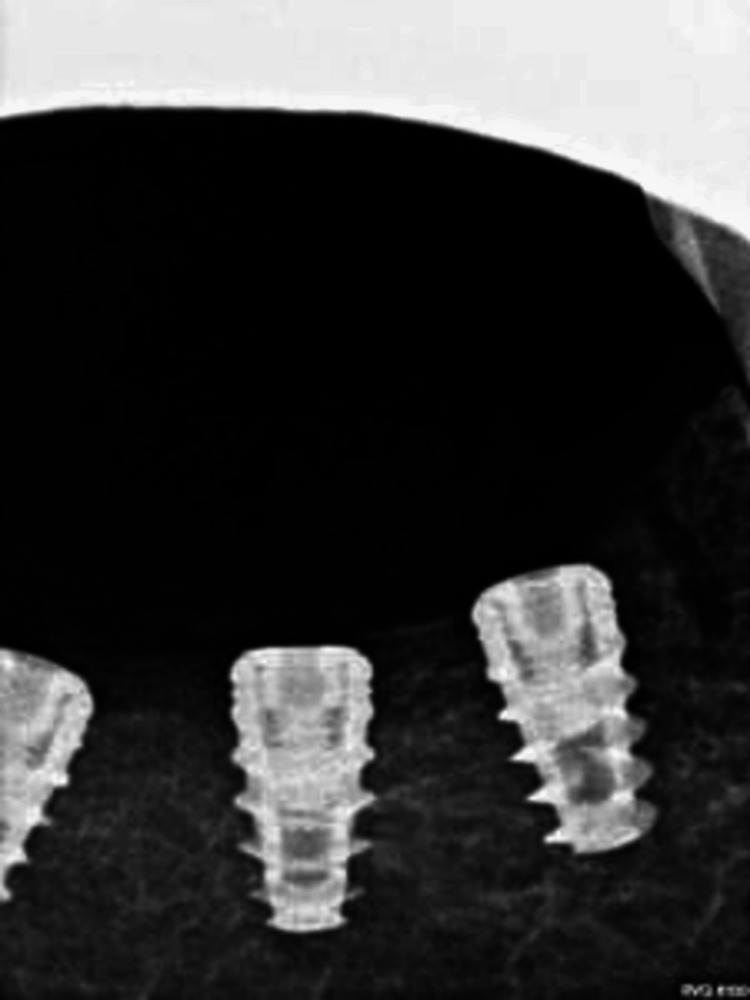
Intraoral periapical radiography of the mandibular region with implants

A postoperative X-ray was done to assess and confirm the proper positioning of the implants placed in the posterior region of the mandible (Figure 7).

**Figure 8:**
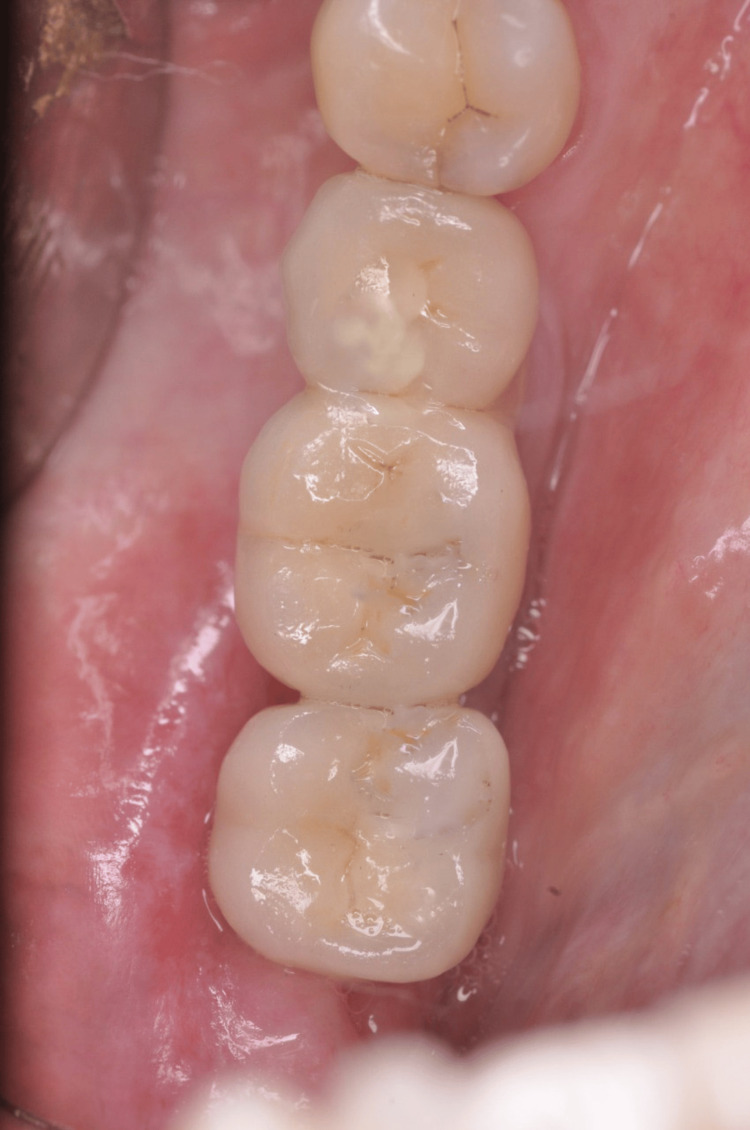
Variation in buccal bone widths and observed cavities near the anchor point

The use of bone grafts was notably lacking in this particular strategy. The study recorded data on the variation in buccal bone widths and the identification of any observable cavities located either proximal or distal to the anchor point. This information is visually shown in Figure 8.

Following each surgical procedure, the patients were instructed to take an antibiotic (500 mg amoxicillin + 125 mg clavulanic acid combination) with a non-steroidal anti-inflammatory drug (400 mg Ibuprofen) three times a day for five days. In addition, it was advised to implement a regimen of chlorhexidine mouthwash to be used three times every day for a duration of 10 consecutive days.

## Discussion

The physiological response that takes place in the alveolar ridge subsequent to tooth extraction, encompassing both resorption and remodeling, is an inherent component of the natural healing mechanism [7]. Following tooth loss, the bone surrounding the vacant socket tends to undergo a phenomenon identified as horizontal resorption to a higher degree than vertical resorption, impacting both the anterior and posterior segments of the socket [8]. The process of resorption leads to a reduction in both the length and breadth of the ridge. The ridge-splitting procedure was specifically developed to tackle this concern by horizontally relocating the cortical plate of the dental ridge, converting a defect with only one wall into a defect with four walls. This modification facilitates enhanced vascularization and bone healing, which facilitates the comprehensive restoration of the expanded defect in a manner akin to that observed in an extraction socket [9]. The ridge-splitting procedure for implant implantation is a widely utilized procedure that has been shown to effectively decrease the overall treatment duration [10]. A study was conducted to investigate the effectiveness of a two-stage ridge-splitting process performed on the lower ridge [10].

The outcomes of two stages of atrophied alveolar ridge extension performed using a sonic-air surgical device were also reported by Agabiti and Botticelli [11]. The degenerating distal parts of the mandible were repaired utilizing a split-thickness flap approach in conjunction with an alveolar ridge broadening procedure conducted in a pair of separate surgical stages. A sonic-air operating device was employed, and during the initial surgical procedure, the sole intervention involved performing basal corticotomies exclusively over the buccal plate. Throughout the subsequent stage, sagittal and vertical osteotomies were done to promote the repositioning of the buccal bone plate. Then, dental implants were placed into the established gap within the buccal and lingual plates. Bone replacements were not utilized, and during the implantation stage, measurements were taken to determine the breadth across the buccal skeletal wall that had been displaced, along with the spaces that had formed both mesially and farther from the implant. The breadth of the alveolar crest was identified on the basis of cone-beam computed tomography (CBCT) scans recorded prior to the initial and subsequent procedures, together with the breadth of the lingering mesial in addition to distal gaps subsequent to implant placement. The study included an overall number of 10 patients, consisting of six females and four men. The age range of the participants was between 37 and 69 years old. During the investigation, a total of 15 implants were implanted in enlarged, thin ridges. From a clinical perspective, the average thickness at the buccal bone wall has been estimated to be 1.2 ± 0.2 millimeters, with gap measurements ranging between 2.8-3.2 millimeters. During the radiographic evaluations, the average starting breadth at the alveolar bone crest was estimated to be 4.1 ± 0.5 millimeters. Following ridge extension, this width increased to 6.8 ± 0.9 millimeters (p<0.01).

In the clinical investigation conducted by Almarrawi, the utilization of a two-stage ridge-splitting procedure was implemented for the purpose of treating individuals presenting with an edentulous narrow alveolar bone ridge in the posterior mandibular area. The findings indicated a notable augmentation in the width of the alveolar bone, with an average bone gain of 2.8 mm post-surgery and 2.22 mm four months post-surgery. After a period of functional loading lasting six months, the implants’ survival rate was found to be 93.3%. Furthermore, it was observed that 84.61% of the enlarged areas successfully achieved a satisfactory width [12].

The investigative report examined a retrospective investigation encompassing a cohort of 13 patients. The individuals in question, who exhibited a total of 17 cases of horizontal deficiencies in the alveolar ridge located at the back of the mandible, underwent a surgical intervention known as the piezoelectric hinge-assisted ridge split technique. After an average recovery period of 14 weeks, dental implants were successfully inserted into the enhanced regions [13].

Clinical case reports presented three instances of substantial horizontal bone deficiency in the posterior region of the mandible. The management of these instances involved implementing the modified alveolar ridge augmentation procedure. The results demonstrated the effectiveness of the approach, particularly in situations where there were limitations in horizontal bone health [14].

This technique may be deemed a secure procedure with satisfactory outcomes when relevant instances are chosen. The attainment of success is contingent upon multiple factors, including the implementation of a biocompatible platform, the meticulous observation of post-operative signs of inflammation, and the assessment of ridge thickness [15-17]. The proper implementation of the surgery is essential for maintaining the stability of the implant and attaining favorable outcomes. While the studies offer promising insights, it is important to take into account the restricted sample sizes and inherent limitations associated with CBCT imaging. The ambiguity surrounding the long-term success of the ridge split technique is attributed to the limited duration of follow-up periods and variations in the documentation of outcomes. Moreover, a narrow focus on surgical results overlooks significant variables, including unique patient attributes and aesthetic considerations.

## Conclusions

The two-stage ridge split technique is emerging as a promising strategy for addressing the challenge of narrow alveolar ridges, particularly in situations where substantial bone volume is imperative to ensure the long-term viability of dental implant procedures. However, a critical exigency exists for more comprehensive and protracted scientific investigations that encompass not only the quantitative outcomes of surgical interventions but also a thorough exploration of qualitative aspects related to patient experiences. Such investigations are indispensable to providing a holistic evaluation of the overall efficacy of this approach within the realm of implant dentistry.

It is imperative to underscore that the efficacy of the ridge split method is contingent upon the meticulous execution of surgical protocols, characterized by precise osteotomies and careful management of soft and hard tissues. Furthermore, the judicious consideration of diverse patient-specific factors, such as bone quality, quantity, and anatomical variations, is pivotal in achieving favorable outcomes with this technique. As the field of implantology continues to advance, comprehensive scientific inquiries are warranted to refine our understanding of the ridge split approach and its applicability in various clinical contexts.
